# Analyzing the Effects of Demographic Differences on Patient Outcomes Following Non-pyogenic Intracranial Venous Thrombosis

**DOI:** 10.7759/cureus.19753

**Published:** 2021-11-19

**Authors:** Kevin F Labagnara, Jessie Birnbaum, Santiago R Unda, David J Altschul

**Affiliations:** 1 Neurological Surgery, Montefiore Medical Center, Bronx, USA

**Keywords:** anemia, primary expected payer, race, non-pyogenic, cerebral venous thrombosis

## Abstract

Objective: To investigate the effect of racial and demographic differences on the short-term outcome of patients following a non-pyogenic cerebral venous thrombosis.

Methods: Data from the National Inpatient Sample were gathered from the years 2013 to 2016. Patients who had a non-pyogenic cerebral venous thrombosis were identified. Admissions of patients between different racial groups were compared. Outcome measures included inpatient mortality, length of stay (LOS), all patients refined diagnosis-related group (APR-DRG) severity and mortality risk scores, non-routine discharges, total charges, sepsis, and urinary tract infections (UTIs).

Results: We identified 973 patients who were admitted with a non-pyogenic cerebral venous thrombosis between 2013 and 2016. Of those, 65.7% were classified as White, 15.6% as Black, 14.1% as Hispanic, and 4.6% as Asian or Pacific Islander. Compared to White patients, Black patients were found to have a higher severity score upon admission (2.94 ± 0.818 vs 2.77 ± 0.839; p = 0.025) as well as a longer adjusted LOS (8.085 ± 5.917 vs 6.503 ± 5.552; p = 0.004) and log LOS (0.934 ± 0.507 vs 0.773 ± 0.521; p = 0.001). On initial analysis, we found that older age, elevated WBC count, income group, anemia, and an expected primary payer of Medicare/Medicaid were significantly associated with Black race and also worse outcomes. When controlling for these variables using multivariate regression, older age, lower income group, and elevated WBC count were not significantly associated with any measures of outcome. The race was no longer associated with a higher APR-DRG severity score but was still significant for adjusted LOS (p = 0.001) and adjusted log LOS (p = 0.004). Lastly, we noted that anemia and the expected primary payer of Medicare/Medicaid were both independently and significantly associated with APR-DRG severity score (p = 0.003; p = 0.010) and the adjusted log LOS (p = 0.019; p = 0.035).

Conclusions: Black patients admitted with a non-pyogenic intracranial venous thrombosis have significantly longer LOS compared to White patients. Anemia and Medicare/Medicaid as the primary expected payer also seem to play a role in longer LOS, as well as the severity of the case.

## Introduction

Cerebral venous thrombosis (CVT) is a rare and often overlooked, yet important, cause of stroke [1]. Although CVT only accounts for 0.5% of strokes [2], it has an estimated mortality rate of 5-10% [1]. CVT can be categorized as non-pyogenic or pyogenic. Pyogenic CVTs are associated with head trauma or disseminated infection from the cranial sinuses, while the non-pyogenic form tends to be multifactorial, influenced by general pathologic conditions that lead to venous thrombosis [3]. Although CVT carries a generally favorable prognosis (~15% of patients remain dependent or die), rapid diagnosis and treatment are essential to favorable patient outcomes [4]. Thus, an understanding of the risk factors and associated conditions can greatly aid clinicians in the early recognition of CVT.

To date, there have been limited studies analyzing the role of racial differences in CVT outcomes. Although they have been described in relation to inherited thrombophilias [5] and associated genetic etiologies [6], only a handful of smaller studies have shown significant differences in outcomes with regards to race and ethnicity [7,8]. In this study, we used the National Inpatient Sample (NIS) data from the years 2013 to 2016 to analyze the effect of race, ethnicity, and structural factors on patient morbidity and mortality in the setting of non-pyogenic venous thrombosis. We expect to find significant differences in outcomes between demographic and lower socioeconomic groups.

## Materials and methods

Study design

This cohort study uses the NIS database for the years 2013-2016. It was deemed exempt from review by the local institutional review board (IRB) because, under Health Insurance Portability and Accountability Act (HIPAA), limited patient datasets are not required to undergo IRB review [9]. The NIS is the largest inpatient database in the United States, comprised of demographic, diagnostic, procedural, and discharge information from over 7 million hospital admissions and approximately 1,000 hospitals per year. The diagnosis and procedural data are captured in the form of the International Classification of Diseases, Ninth Revision (ICD9) codes for the years 2013, 2014, and the first through third quarters of 2015. The International Classification of Diseases, Tenth Revision (ICD10) codes are used for the fourth quarter of 2015 and the full year of 2016.

Patient selection

Primary inclusion criteria for our study were patients who had a diagnosis of a non-pyogenic thrombosis of the intracranial venous system. Patients were identified by using the ICD9 code for “nonpyogenic thrombosis of intracranial venous sinus” (437.6), the ICD10 code for “nonpyogenic thrombosis of intracranial venous system” (I67.6), and the ICD10 code for “cerebral infarction due to cerebral venous thrombosis, nonpyogenic” (I63.6). The ICD9 code for “phlebitis and thrombophlebitis of intracranial venous sinuses” (325) was considered but ultimately not included as it does not differentiate pyogenic from nonpyogenic. Together these codes sufficiently identified our patients of interest while excluding others that did not qualify. These codes were identified and verified from the WHO website.

Data collection

Patient data that were analyzed included patient age, sex, race, day of admission, expected primary payer, income group, hospital teaching status, and comorbidities. Patient race was classified as White, Black, Hispanic, and Asian/Pacific Islander, while those categorized as Native American or other were excluded from the analysis, due to sample size limitations. The income group was divided into four brackets using the median income of their zip code, which were subsequently adjusted for inflation each year. The 2013-2016 ranges for each income bracket are as follows: ~$40,000 and below, ~$40,000-$50,000, ~$50,000-$65,000, and ~$65,000 and above. The expected primary payer was classified as Medicare/Medicaid or private insurance while excluding other methods of payment due to their limited number. Patients were not separated into Medicare vs Medicaid due to its association with age. Hospital teaching status was divided into teaching (urban teaching) and non-teaching (rural and urban non-teaching), as rural teaching hospitals are rare. Comorbidities analyzed included hypertensive diseases, elevated WBC count (unspecified), hypoosmolarity/hyponatremia, acidosis, overweight/obesity, diabetes mellitus type 2 (with and without complications), current or former smoker, and anemia. For patients who were under the ICD9 codes, there were pre-set variables for their comorbidities. For patients under ICD10, comorbidities were deduced from their concurrent ICD codes which are similar to how the ICD9 comorbidities were initially classified.

Outcome data

Outcome measures analyzed were mortality, length of stay (LOS), adjusted LOS, all patients refined diagnosis-related group (APR-DRG) severity and risk of mortality, total charges, non-routine discharges (discharge other than home, self-care, or planned acute care hospital inpatient admission), and complications (urinary tract infections and sepsis).

The APR-DRG severity and risk of mortality are scores from 1 to 4 that represent the patient’s condition at the time of discharge and include the impact of conditions that developed during the hospital stay. The APR-DRG severity score refers to the extent of physiologic decompensation or organ system loss of function and is ranked from 1 to 4 as minor, moderate, major, and extreme loss of function. APR-DRG risk of mortality score refers to the likelihood of dying and is ranked from 1 to 4 as minor, moderate, major, and the extreme likelihood of dying [10].

A non-routine discharge is classified as a discharge other than to home, self-care, or planned acute care hospital inpatient admission. This may include skilled nursing facilities, intermediate care facilities, or short-term hospitals.

All continuous outcome variables were assessed for normal distribution before analysis. As a result, the LOS variable was modified into two similar measures to fit a more normal distribution, which were both ultimately used in analysis to reduce the effect of confounders on hospital stay. The first measure, called “adjusted LOS,” excluded patients with mortality during their hospital stay and also excluded those who stayed over 28 days. The second LOS measure, called “adjusted log LOS,” excluded patients with mortality during their hospital stay and then took the logarithm 10 of the remaining data points.

Statistical analysis

All statistical analyses were conducted in IBM SPSS Statistics v.25 (IBM SPSS Statistics, Armonk, NY). Categorical variables were described using frequencies and proportions and compared using χ2 tests. Continuous values were expressed using mean ± standard deviation (SD) and compared using Student’s t-test or the non-parametric Mann-Whitney test as appropriate. A p-value of less than 0.05 was considered statistically significant.

Variables with a p-value of less than <0.1 in the univariate analysis were then included in the multivariable models. For the multivariable analysis, we utilized linear regression with three models. Model 1 analyzed the effect of race on the outcomes of interest, Model 2 then added demographic variables found to be significantly associated with race in the univariate analysis, and Model 3 additionally included comorbidities found to be significantly associated with race.

## Results

A total of 973 patients with a non-pyogenic thrombosis of the intracranial venous system between the years 2013 and 2016 were identified using the ICD10 codes I67.6 and I63.6, along with the ICD9 code of 437.6. Demographically, 65.7% were classified as White, 15.6% as Black, 14.1% as Hispanic, and 4.6% as Asian or Pacific Islander. The mean age (±SD) of the patients was 46.7 ± 22.5 years, and 57.9% of patients were female. Of patients, 21.7% were admitted on a weekend day (Saturday/Sunday) and 85.7% of patients were admitted to a teaching hospital. The expected primary payer for 55.8% was Medicare or Medicaid and was private insurance for the other 44.2%.

There were significant differences noted in mean age, income group, and expected primary payer between racial groups (Table 1). In regard to comorbidities, there were significant differences for elevated WBC count, anemia, and sickle cell disorders between racial groups (Table 1). These differences are accounted for via multivariate regression and are discussed further in this section. Sickle cell disorders were not included in the further analysis due to insufficient sample size.

**Table 1:** Baseline characteristics of patients admitted with a diagnosis of non-pyogenic intracranial venous thrombosis between 2013 and 2016. * p-value below 0.05; ICH, intracerebral hemorrhage.

Parameter	All patients	White	Black	Hispanic	Asian/Pacific Islander	P-value
Number of cases	973	639	152	137	45	
Age, years, mean ± SD	46.70 ± 22.497	49.21 ± 22.752	40.90 ± 20.911	41.28 ± 21.448	47.09 ± 21.285	<0.001*
Gender	973					0.075*
Male (count, %)	410 (42.1%)	280 (43.8%)	50 (32.9%)	58 (42.3%)	22 (48.9%)	
Female (count, %)	563 (57.9%)	359 (56.2%)	102 (67.1%)	79 (57.7%)	23 (51.1%)	
Income groups	951					<0.001*
Quartile 1	243 (25.5%)	128 (20.4%)	59 (40.4%)	51 (38.1%)	5 (11.4%)	
Quartile 2	225 (23.7%)	153 (24.4%)	30 (20.5%)	35 (26.1%)	7 (15.9%)	
Quartile 3	249 (26.2%)	178 (28.4%)	33 (22.6%)	28 (20.9%)	10 (22.7%)	
Quartile 4	234 (24.6%)	168 (26.8%)	24 (16.4%)	20 (14.9%)	22 (50.0%)	
Weekend (count, %)	973					0.512
Weekend	211 (21.7%)	133 (20.8%)	40 (26.3%)	29 (21.2%)	9 (20.0%)	
Weekday	762 (78.3%)	506 (79.2%)	112 (73.7%)	108 (78.8%)	36 (80.0%)	
Expected primary payer (count, %)	884					0.046*
Medicare/Medicaid	493 (55.8%)	311 (52.9%)	85 (63.9%)	76 (62.3%)	21 (51.2%)	
Private	391 (44.2%)	277 (47.1%)	48 (36.1%)	46 (37.7%)	20 (48.8%)	
Teaching status (count, %)	973					0.645
Teaching	834 (85.7%)	542 (84.4%)	135 (88.8%)	118 (86.1%)	39 (86.7%)	
Non-teaching	139 (14.3%)	97 (15.2%)	17 (11.2%)	19 (13.9%)	6 (13.3%)	
Comorbidities (count, %)						
Hypertensive diseases	415 (42.7%)	274 (42.9%)	64 (42.1%)	62 (45.3%)	15 (33.3%)	0.570
Elevated WBC count	47 (4.8%)	37 (5.8%)	5 (3.3%)	1 (0.7%)	4 (8.9%)	0.034*
Hypoosmolarity/hyponatremia	33 (3.4%)	25 (3.9%)	5 (3.3%)	3 (2.2%)	0 (0.0%)	0.437
Acidosis	60 (6.2%)	38 (5.9%)	9 (5.9%)	11 (8.0%)	2 (4.4%)	0.772
Overweight/obese	112 (11.5%)	71 (11.1%)	23 (15.1%)	16 (11.7%)	2 (4.4%)	0.234
Diabetes type 2	147 (15.1%)	99 (15.5%)	22 (14.5%)	22 (16.1%)	4 (8.9%)	0.665
Current or former smoker	249 (25.6%)	170 (26.6%)	33 (21.7%)	36 (26.3%)	10 (22.2%)	0.604
Anemia	191 (19.6%)	100 (15.6%)	48 (31.6%)	32 (23.4%)	11 (24.4%)	<0.001*
Sickle cell disorders	13 (1.3%)	4 (0.6%)	8 (5.3%)	1 (0.7%)	0 (0.0%)	0.000
Cerebral edema	171 (17.6%)	117 (18.3%)	28 (18.4%)	15 (10.9%)	11 (6.4%)	0.115
Cerebral infarction	220 (22.6%)	142 (22.2%)	39 (25.7%)	28 (20.4%)	11 (24.4%)	0.725
Nontraumatic ICH	134 (13.8%)	95 (14.9%)	19 (12.5%)	14 (10.2%)	6 (13.3%)	0.509
Hydrocephalus	39 (4%)	23 (3.6%)	6 (3.9%)	6 (4.4%)	4 (8.9%)	0.374
Malignancy	104 (10.7%)	72 (11.3%)	12 (7.9%)	14 (10.2%)	6 (13.3%)	0.609
Nicotine dependence	132 (13.6%)	90 (14.1%)	18 (11.8%)	18 (13.1%)	6 (13.3%)	0.907
Alcohol-related disorders	53 (5.4%)	38 (5.9%)	7 (4.6%)	6 (4.4%)	2 (4.4%)	0.823
Hormonal contraceptives	11 (1.1%)	8 (1.3%)	2 (1.3%)	1 (0.7%)	0 (0.0%)	0.839

Table 2 shows the differences in patient measures of the outcome when comparing racial groups for mortality, APR-DRG risk of mortality score, APR-DRG severity score, log LOS, adjusted LOS, total charges, non-routine discharges, rates of sepsis, and rate of UTIs. There were significant differences (p < 0.05) observed between White and Black patients for APR-DRG severity score (2.77 ± 0.839 vs 2.94 ± 0.818), log LOS (0.773 ± 0.521 vs 0.934 ± 0.507), and adjusted LOS (6.503 ± 5.552 vs 8.085 ± 5.917). Although the other outcome measures did not fall below the 0.05 significance threshold, the means were elevated for nearly every negative outcome measure when comparing Black to White patients. When comparing Hispanic and Asian/Pacific Islander patients to White patients, the measures of outcome were mixed but mostly not significant. The only significant measure of outcome was for total charges ($116,639 ± 199,860 vs $185,851 ± 282,288) between White and Asian/Pacific Islander patients but will not be discussed further due to the limited sample size.

**Table 2:** Outcome measures of mortality, APR-DRG severity score, APR-DRG risk of mortality score, non-routine discharge, adjusted log LOS, adjusted LOS, total charges, urinary tract infections, and sepsis between racial groups. * p-value below 0.05; APR-DRG, all patients refined diagnosis-related group; LOS, length of stay; UTI, urinary tract infection.

Parameter	All patients	White	Black	Hispanic	Asian/Pacific Islander	P-value
Number of cases	973	639 (65.7%)	152 (15.6%)	137 (14.1%)	45 (4.6%)	
Mortality (%)	46 (4.7%)	32 (5.0%)	8 (5.3%)	5 (3.6%)	1 (2.2%)	0.756
APR-DRG severity score (mean ± SD)	2.81 ± 0.842	2.77 ± 0.839	2.94 ± 0.818 (p = 0.025*)	2.81 ± 0.862 (p = 0.611)	2.91 ± 0.874 (p = 0.277)	0.126
APR-DRG mortality risk (mean ± SD)	2.35 ±1.124	2.32 ± 1.119	2.48 ± 1.165 (p = 0.129)	2.30 ± 1.074 (p = 0.827)	2.51 ± 1.119 (p = 0.277)	0.320
Non-routine discharge (%)	400 (41.2%)	265 (41.5%)	65 (42.8%)	48 (35.0%)	22 (48.9%)	0.330
Adjusted log LOS (mean ± SD)	0.803 ± 0.515	0.773 ± 0.521	0.934 ± 0.507 (p = 0.001*)	0.787 ± 0.484 (p = 0.773)	0.823 ± 0.492 (p = 0.527)	0.006
Adjusted LOS (mean ± SD)	6.782 ± 5.615	6.503 ± 5.552	8.085 ± 5.917 (p = 0.004*)	6.738 ± 5.442 (p = 0.669)	6.775 ± 5.682 (p = 0.767)	0.037
Total charges (mean ± SD)	$122,821 ± 212,031	$116,639 ± 199,860	$129,360 ± 251,872 (p = 0.510)	$125,773 ± 192,996 (p = 0.654)	$185,851 ± 282,288 (p = 0.045*)	0.237
UTIs	75 (7.7%)	49 (7.7%)	10 (6.6%)	12 (8.8%)	4 (8.9%)	0.902
Sepsis	40 (4.1%)	29 (4.5%)	8 (5.3%)	2 (1.5%)	1 (2.2%)	0.301

We next utilized multivariable regression to observe whether the variables in Table 1 that were significantly associated with race had an independent effect on the measures of outcome (APR-DRG severity score, adjusted log LOS, and adjusted LOS). Dummy variables were created for variables that had more than two groups within them such as race and income group.

Upon analyzing the effect of these variables on the APR-DRG severity score, we found that race was no longer significant (p = 0.079) when controlling for the demographic variables and comorbid conditions (Table 3). In the final model, we instead discovered that the female gender (p = 0.007) was inversely related, while Medicare/Medicaid as the primary payer (p = 0.040) and anemia (p = 0.004) were directly related to the APR-DRG severity score (Table 4). This suggests that the true difference in APR-DRG severity score is due to gender, the expected primary payer, and anemia.

**Table 3:** Multivariable linear regression for APR-DRG severity score. Model 1 is race, Model 2 adds demographic factors (age, gender, income, and primary expected payer), and Model 3 adds comorbidities (elevated WBCs and anemia). * p-value below 0.05; APR-DRG, all patients refined diagnosis-related group.

Multivariable analysis	Model 1 (unadjusted)	Model 2 (Model 1 + demographics)	Model 3 (Model 2 + comorbidities)
P-value	0.036*	0.032*	0.079
Unstandardized coefficient (95% confidence interval)	0.174 (0.012-0.336)	0.182 (0.015-0.349)	0.150 (-0.018-0.318)

**Table 4:** Multivariable linear regression for APR-DRG severity score. Model 1 is race, Model 2 adds demographic factors (age, gender, income, and primary expected payer), and Model 3 adds comorbidities (elevated WBCs and anemia). * p-value below 0.05; APR-DRG, all patients refined diagnosis-related group.

Model	Variable	Unstandardized coefficient (B)	Lower bound CI (95%)	Upper bound CI (95%)	Significance
1	Constant	2.763	2.694	2.832	
	Race (Black)	0.174	0.012	0.336	0.036*
	Race (Hispanic)	0.027	-0.140	0.194	0.751
	Race (Asian/Pacific Islander)	0.237	-0.034	0.508	0.086
2	Constant	2.713	2.527	2.899	
	Race (Black)	0.182	0.015	0.349	0.032
	Race (Hispanic)	0.020	-0.149	0.190	0.813
	Race (Asian/Pacific Islander)	0.250	-0.021	0.521	0.070
	Age	0.001	-0.002	0.003	0.701
	Gender (Female)	-0.159	-0.275	-0.043	0.007*
	Income (Quartile 1)	0.061	-0.103	0.225	0.464
	Income (Quartile 2)	0.089	-0.076	0.253	0.290
	Income (Quartile 3)	0.071	-0.086	0.227	0.375
	Expected primary payer (Medicare/Medicaid)	0.120	-0.001	0.241	0.071
3	Constant	2.679	2.493	2.866	
	Race (Black)	0.150	-0.018	0.318	0.079
	Race (Hispanic)	0.005	-0.164	0.175	0.950
	Race (Asian/Pacific Islander)	0.230	-0.040	0.501	0.095
	Age	0.000	-0.002	0.003	0.780
	Gender (Female)	-0.160	-0.276	-0.044	0.007*
	Income (Quartile 1)	0.062	-0.102	0.225	0.460
	Income (Quartile 2)	0.083	-0.081	0.246	0.322
	Income (Quartile 3)	0.075	-0.081	0.231	0.348
	Expected primary payer (Medicare/Medicaid)	0.127	0.006	0.247	0.040*
	Elevated WBC count	0.062	-0.198	0.323	0.639
	Anemia	0.210	0.068	0.352	0.004*

When analyzing the effect of the demographic and comorbid variables associated with race on the adjusted log LOS, we found that race remained significant (p = 0.004) in the final model when controlling for these variables (Table 5). We also discovered that female gender (p = 0.017) was again inversely related, while Medicare/Medicaid as the primary payer (p = 0.026) and anemia (p = 0.020) were directly related with the adjusted log LOS (Table 6). This indicates a role for each in prolonging the patient’s hospital course.

**Table 5:** Multivariable linear regression for adjusted log LOS. Model 1 is race, Model 2 adds demographic factors (age, gender, income, and primary expected payer), and Model 3 adds comorbidities (elevated WBCs and anemia). * p-value below 0.05; LOS, length of stay.

Multivariable analysis	Model 1 (unadjusted)	Model 2 (Model 1 + demographics)	Model 3 (Model 2 + comorbidities)
P-value	0.001*	0.002*	0.004*
Unstandardized coefficient (95% confidence interval)	0.165 (0.066-0.264)	0.163 (0.062-0.265)	0.149 (0.046-0.251)

**Table 6:** Multivariable linear regression for adjusted log LOS. Model 1 is race, Model 2 adds demographic factors (age, gender, income, and primary expected payer), and Model 3 adds comorbidities (elevated WBCs and anemia). * p-value below 0.05; LOS, length of stay.

Model	Variable	Unstandardized coefficient (B)	Lower bound CI (95%)	Upper bound CI (95%)	Significance
1	Constant	0.774	0.732	0.815	
	Race (Black)	0.165	0.066	0.264	0.001*
	Race (Hispanic)	0.004	-0.097	0.106	0.931
	Race (Asian/Pacific Islander)	0.062	-0.103	0.227	0.460
2	Constant	0.804	0.691	0.917	
	Race (Black)	0.163	0.062	0.265	0.002*
	Race (Hispanic)	-0.007	-0.110	0.096	0.892
	Race (Asian/Pacific Islander)	0.064	-0.101	0.228	0.449
	Age	-0.001	-0.002	0.001	0.352
	Gender (female)	-0.086	-0.156	-0.015	0.017*
	Income (Quartile 1)	0.001	-0.099	0.101	0.980
	Income (Quartile 2)	0.078	-0.022	0.178	0.128
	Income (Quartile 3)	-0.012	-0.108	0.083	0.799
	Expected primary payer (Medicare/Medicaid)	0.080	0.006	0.153	0.034*
3	Constant	0.787	0.673	0.900	
	Race (Black)	0.149	0.046	0.251	0.004*
	Race (Hispanic)	-0.013	-0.116	0.091	0.812
	Race (Asian/Pacific Islander)	0.053	-0.111	0.218	0.526
	Age	-0.001	-0.002	0.001	0.292
	Gender (Female)	-0.086	-0.156	-0.015	0.017*
	Income (Quartile 1)	0.000	-0.099	0.100	0.996
	Income (Quartile 2)	0.074	-0.026	0.174	0.146
	Income (Quartile 3)	-0.010	-0.105	0.085	0.832
	Expected primary payer (Medicare/Medicaid)	0.084	0.010	0.157	0.026*
	Elevated WBC count	0.069	-0.090	0.228	0.397
	Anemia	0.103	0.016	0.189	0.020*

Lastly, when analyzing the effect of the demographic and comorbid variables associated with the race on the adjusted LOS, we found that race remained significant (p = 0.015) in the final model (Table 7). Additionally, older age (p = 0.05) and being a part of the second-lowest income quartile (p = 0.015) were noted to significantly increase the adjusted LOS (Table 8). This indicates an independent role for each in prolonging the patient’s hospital course.

**Table 7:** Multivariable linear regression for adjusted LOS. Model 1 is race, Model 2 adds demographic factors (age, gender, income, and primary expected payer), and Model 3 adds comorbidities (elevated WBCs and anemia). * p-value below 0.05; LOS, length of stay.

Multivariable analysis	Model 1 (Unadjusted)	Model 2 (Model 1 + demographics)	Model 3 (Model 2 + comorbidities)
P-value	0.003*	0.012*	0.015*
Unstandardized coefficient (95% confidence interval)	1.728 (0.593-2.863)	1.508 (0.339-2.678)	1.462 (0.281-2.644)

**Table 8:** Multivariable linear regression for adjusted LOS. Model 1 is race, Model 2 adds demographic factors (age, gender, income, and primary expected payer), and Model 3 adds comorbidities (elevated WBCs and anemia). * p-value below 0.05; LOS, length of stay.

Model	Variable	Unstandardized coefficient (B)	Lower bound CI (95%)	Upper bound CI (95%)	Significance
1	Constant	6.429	5.963	6.896	
	Race (Black)	1.728	0.593	2.863	0.003*
	Race (Hispanic)	0.057	-1.074	1.187	0.921
	Race (Asian/Pacific Islander)	0.765	-1.086	2.617	0.418
2	Constant	6.778	5.498	8.058	
	Race (Black)	1.508	0.339	2.678	0.012*
	Race (Hispanic)	-0.274	-1.428	0.881	0.642
	Race (Asian/Pacific Islander)	0.906	-0.948	2.759	0.338
	Age	-0.018	-0.036	0.001	0.063
	Gender (female)	-0.737	-1.530	0.056	0.068
	Income (Quartile 1)	0.737	-0.387	1.861	0.199
	Income (Quartile 2)	1.420	0.296	2.543	0.013*
	Income (Quartile 3)	0.490	-0.657	1.475	0.452
	Expected primary payer (Medicare/Medicaid)	0.730	-0.105	1.565	0.087
3	Constant	6.652	5.367	7.937	
	Race (Black)	1.462	0.281	2.644	0.015*
	Race (Hispanic)	-0.280	-1.440	0.880	0.636
	Race (Asian/Pacific Islander)	0.852	-1.028	2.678	0.382
	Age	-0.019	-0.037	0.000	0.050*
	Gender (Female)	-0.748	-1.541	0.045	0.064
	Income (Quartile 1)	0.708	-0.416	1.832	0.217
	Income (Quartile 2)	1.387	0.265	2.510	0.015*
	Income (Quartile 3)	0.422	-0.642	1.487	0.436
	Expected primary payer (Medicare/Medicaid)	0.740	-0.095	1.574	0.082
	Elevated WBC count	0.960	-0.848	2.768	0.298
	Anemia	0.803	-0.190	1.796	0.113

## Discussion

The present study examines the possible effects of racial/ethnic factors and various social determinants of health for patients admitted with a non-pyogenic CVT. For the 973 patients we sampled from 2013 to 2016, the overall in-hospital mortality rate was 4.7%, the average adjusted LOS was 6.8 days, and the average APR-DRG severity and risk of mortality scores ranged between moderate and major. Our analysis focused on studying the differences with regard to outcomes following non-pyogenic CVT. To date, there have been few studies with limited subjects looking at this association. Our study utilized the large sample size of the NIS inpatient database to better analyze these variables.

Initially, we found significant differences in APR-DRG severity score (2.94 ± 0.818 vs 2.77 ± 0.839; p = 0.025), adjusted LOS (8.085 ± 5.917 vs 6.503 ± 5.552; p = 0.004), and adjusted log LOS (0.934 ± 0.507 vs 0.773 ± 0.521; p = 0.001) following a non-pyogenic CVT between Black and White patients. There were no significant differences in mortality rates between all races. We then analyzed the demographic and risk factors that were significantly associated with these racial differences to address any potential confounding effects they may have on measures of outcome. These variables were age, income groups, expected primary payer, increased WBC count, and anemia. When controlling for these variables, age and increased WBC were not significantly associated with any measures of outcome.

Race, expected primary payer, anemia, and income group were all significantly associated with one or more measures of outcome; thus we utilized multivariable regression to address if any of them were potentially confounding variables. We first analyzed racial differences in our regression model and noted that while it no longer had a significant effect on the APR-DRG severity score (p = 0.091), it was significant for differences between Black and White patients in the adjusted LOS (p = 0.001) and adjusted log LOS (p = 0.004). We then noted that anemia and the expected primary payer of Medicare/Medicaid were both independently and significantly associated with APR-DRG severity score (p = 0.003; p = 0.010) and the adjusted log LOS (p = 0.019; p = 0.035). Lastly, we found that the income group was no longer significant on the adjusted LOS when controlled for via regression. These results indicate an independent relationship for the race, expected primary payer, male gender, and anemia in prolonging hospital courses, while expected primary payer, male gender, and anemia have a greater role in determining the severity of cases.

While anemia [11-13] and race [7,8] have previously been identified as risk factors for poor outcomes following CVT, we highlight a potentially novel role for the expected primary payer. Although Medicare patients have been previously noted to have higher readmission rates [14], we now see that Medicare patients also have increased severity of disease at the time of presentation and longer hospital LOS. Additionally, our study is the first of its kind to investigate race and CVT outcomes utilizing the large sample size of the NIS inpatient database, allowing us to report significant results with greater confidence. This association between race and socioeconomic factors, such as patient insurance on outcomes following CVT, warrants further research to better elucidate their effects. Our findings represent yet another area in which race/ethnicity and social determinants of health significantly impact outcomes for patients. To negate these disparities, we must first understand the mechanisms by which they have come to pass. These findings elucidate the need for further research looking beyond simply race as a risk factor for poor patient outcome, to the systemic and social frameworks, such as insurance coverage and socioeconomic status, to identify cost-effective and culturally sensitive interventions, which may be implemented at multiple societal levels to improve outcomes for all patients.

While anemia is a well-established risk factor for CVT, the causality of this relationship is unknown and the association between anemia and African descent must be noted. Anemia may be an independent risk factor for CVT [14]; however, it may also be an epidemiologic phenomenon secondary to other prothrombotic factors. Potential causes for this discrepancy include an increased incidence of sickle cell disease and thalassemias among Blacks of African descent. Anemia was included in our analysis due to this association; however, this potential confounder was not able to be further evaluated due to a sample size limitation. While the incidence of all causes of anemia was over two-fold higher in Black patients when compared to White patients, multivariate analysis showed that anemia is a risk factor for prolonged LOS regardless of demographic factors. Further research is warranted to investigate individual causes of anemia in relation to CVT, as our dataset did not include enough patients to make this analysis feasible.

It should be noted that previous studies have found a significant association between lower socioeconomic status as well as the primary payer and rates of leaving against medical advice (AMA) [14]. Patients who leave AMA have been shown to have a 40% increase in one-year-morality and a higher risk of readmission [14]. This association was not controlled for in our analysis and represents a potential limitation to our study. Lastly, the interplay of multiple socioeconomic factors aside from income and insurance, such as occupation, housing, nutrition, education, and others play into patient health, which is not able to be accounted for in this study. As more of these types of variables become incorporated into the NIS and other large medical databases, we will be able to elucidate which of them are associated with patient outcomes.

## Conclusions

Non-pyogenic cerebral venous thrombosis is a rare cause of stroke with high morbidity, mortality, and disease burden. While the pathogenesis of this disease has been thoroughly studied, the role that socioeconomic, structural, and racial factors play in the prognosis of this disease has been largely overlooked up to this point. The findings of our study demonstrate a significant relationship between race, expected primary payer, gender, and anemia in increasing the severity and prolonging the length of stay of patients admitted with a non-pyogenic CVT.

These findings are in line with previous studies analyzing the effects of race and anemia in patient outcomes following a CVT and on those studying expected primary payer on patient readmission. Given our relatively large sample size, our study was able to differentiate the association of race from the potentially confounding effect of anemia. Additionally, we identified a novel correlation for the expected primary payer on the severity of patient presentation and their length of hospital stay. These findings suggest that special consideration should be given to patients admitted with these risk factors to reduce the risk of adverse outcomes.
